# Supplementary material for: Quantification of gallium cryo-FIB milling damage in biological lamellae
Source: Proc Natl Acad Sci U S A. 2023 May 22;120(23):e2301852120. doi: 10.1073/pnas.2301852120 (PMC10266028; doi:10.1073/pnas.2301852120)
Supplement: Supplementary file 1 — Appendix 01 (PDF) [file pnas.2301852120.sapp.pdf]

Supporting Information for

Quantification of gallium cryo-FIB milling damage in biological lamellae.

Bronwyn A. Lucas and Nikolaus Grigorieff

Please send correspondence to
Email: bronwynlucas@berkeley.edu, niko@grigorieff.org

This PDF file includes:

Figures S1 to S6
Tables S1

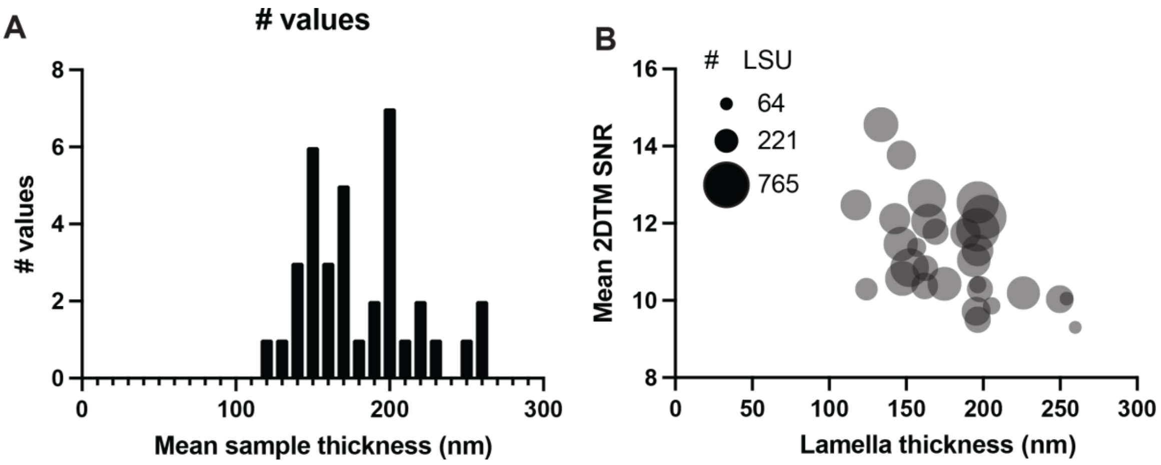

Fig. S1. (A) Histogram showing the distribution of lamella thickness calculated using the Beer-Lambert law. (B) Scatterplot showing the mean 2DTM SNR of the detected LSUs in each image as a function of local lamella thickness. The point size indicates the number of LSUs detected in the image.

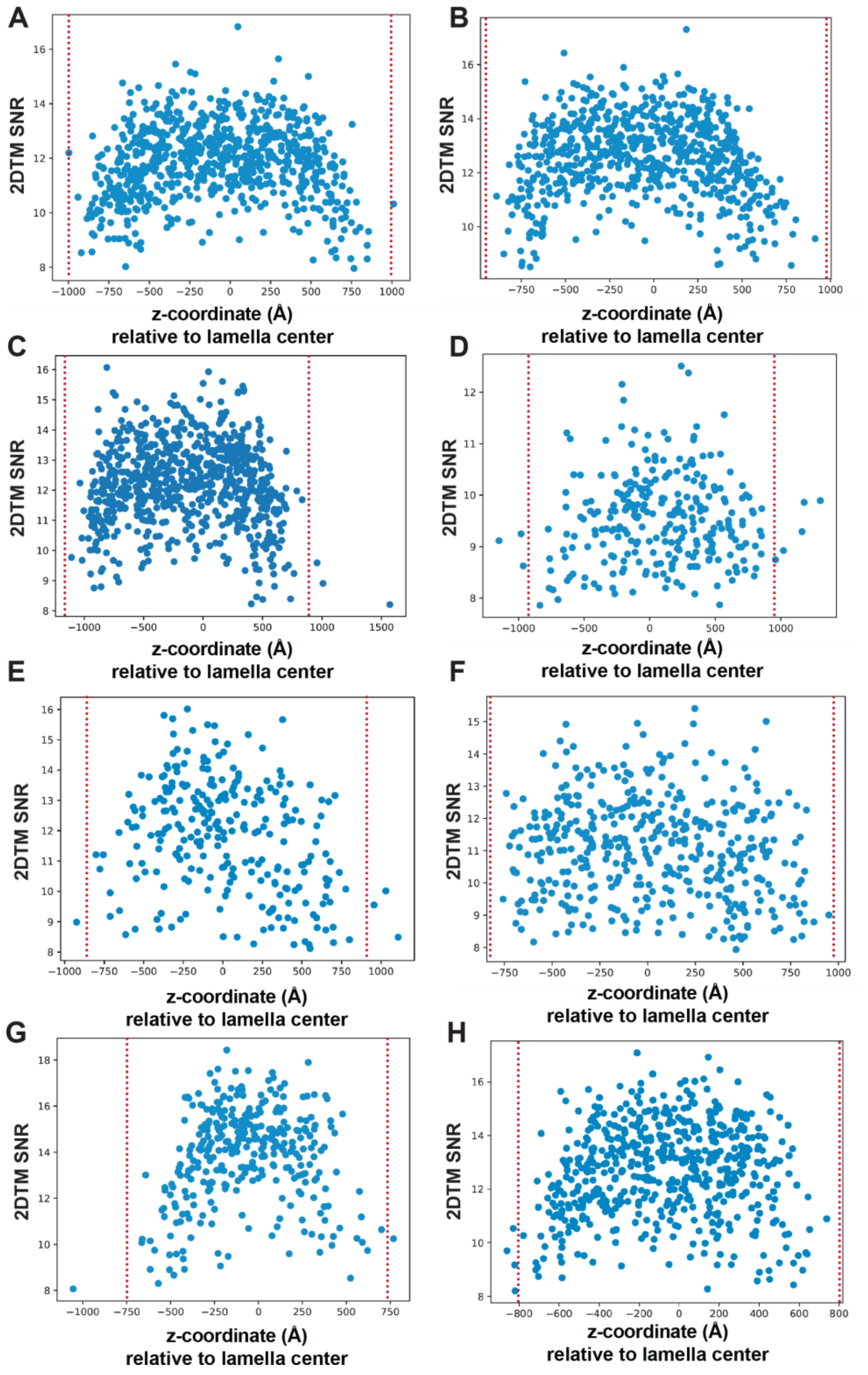

Fig. S2. Representative plots showing the relationship between lamella z-coordinate and 2DTM SNR of LSU-detected targets in FIB-milled yeast lamellae. The dotted red lines indicate the estimated locations of the lamella surfaces, based on average lamella thickness and median z-coordinates as lamella center.

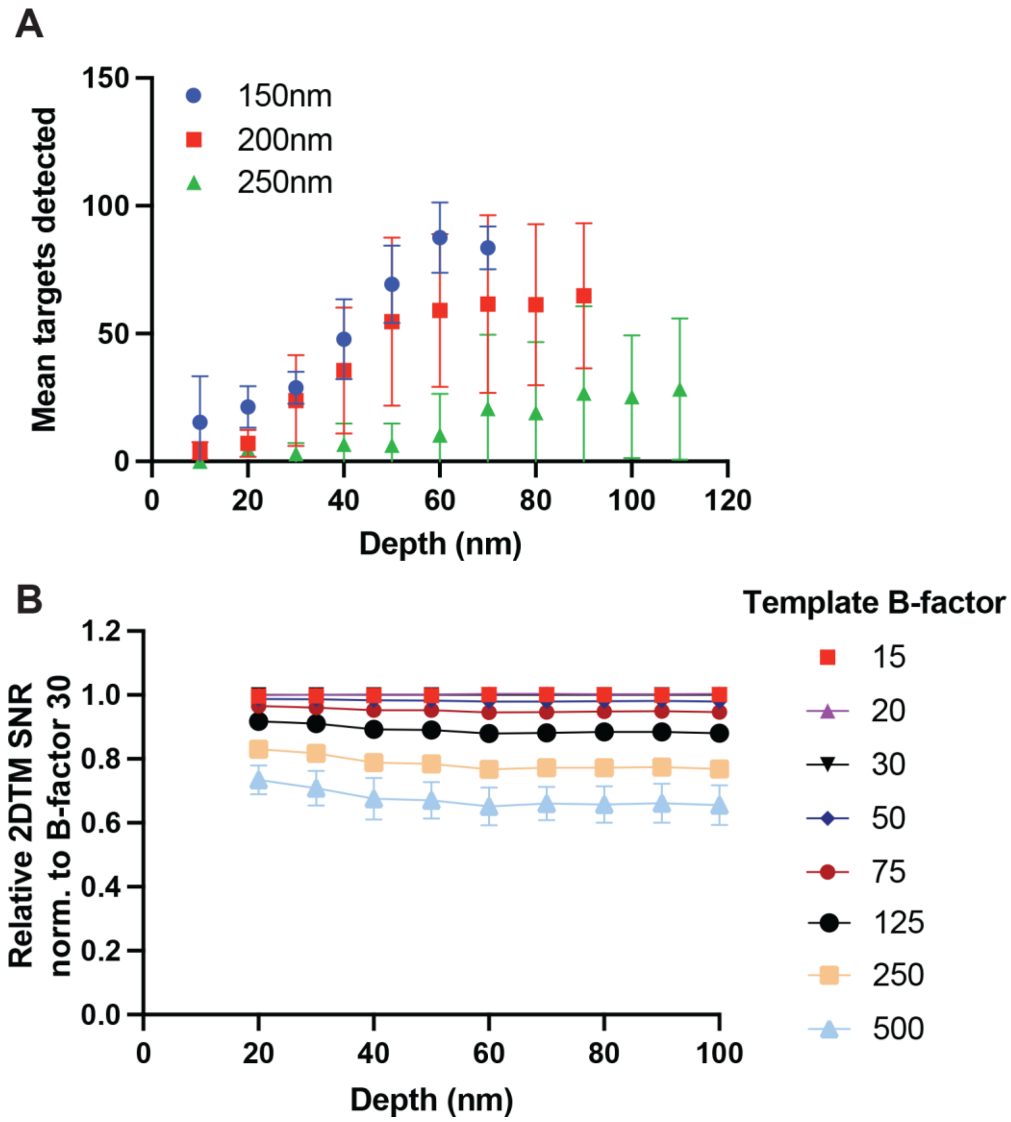

Fig. S3. (A) Scatterplot showing the mean number of targets detected in each lamella z-coordinate bin as a function of depth relative to the nearest lamella surface for lamellae of the indicated thickness. Error bars indicate the standard deviation. (B) Scatterplot showing the change in 2DTM SNR when the B-factor applied to the template is varied as a function of distance from the nearest lamella surface. Values are shown relative to the 2DTM SNR with a B-factor of 30 applied to the template, which corresponds to the B-factor applied to the template to localize targets.

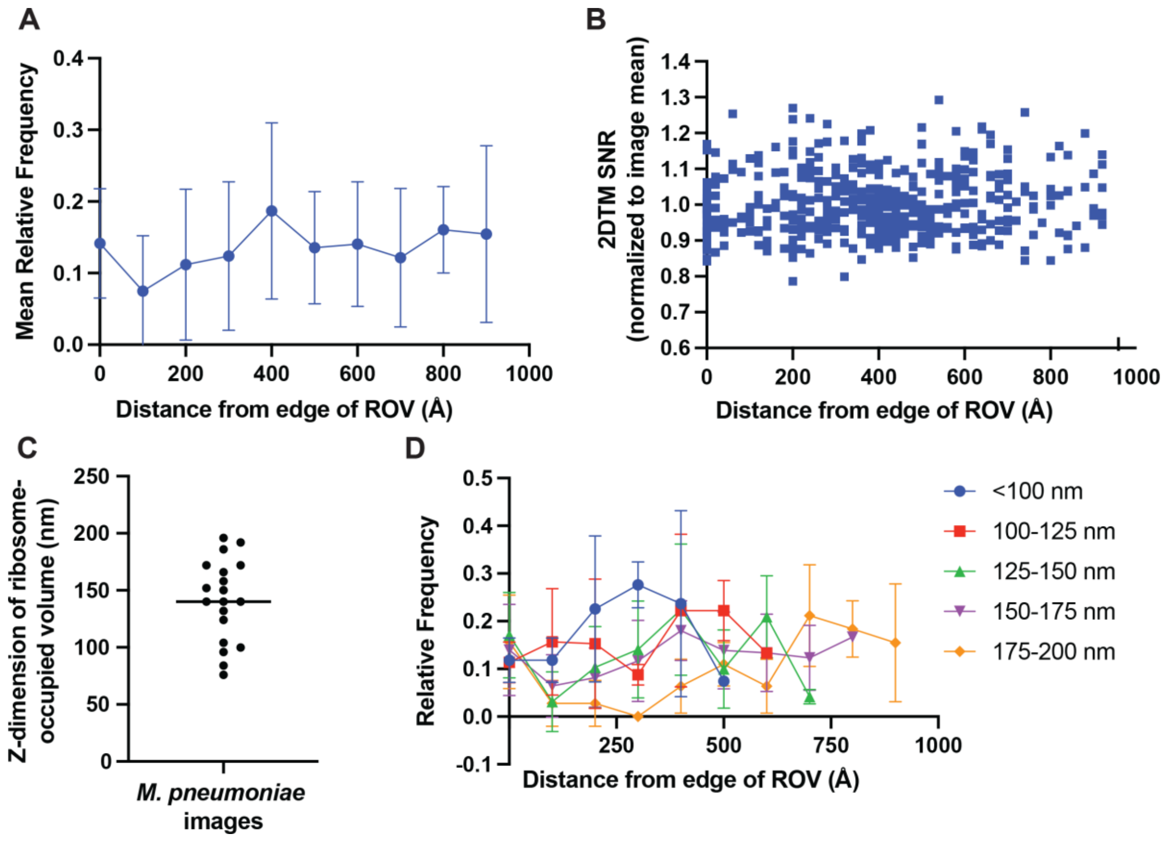

Fig. S4. No consistent relationship between 2DTM SNRs and depth in unmilled *Mycoplasma pneumoniae* cells. (A) Scatterplot showing the mean proportion of LSUs identified in each bin as a function of distance from the edge of the ribosome occupied volume (ROV). (A) Scatterplot showing the 2DTM SNR relative to the image mean for each identified LSU as a function of distance from the edge of the ROV. (C) Plot showing the z-dimension of the ROV in images of *Mycoplasma pneumoniae* cells. (D) As in A), showing the results grouped by thickness.

M. pneumoniae unmilled samples

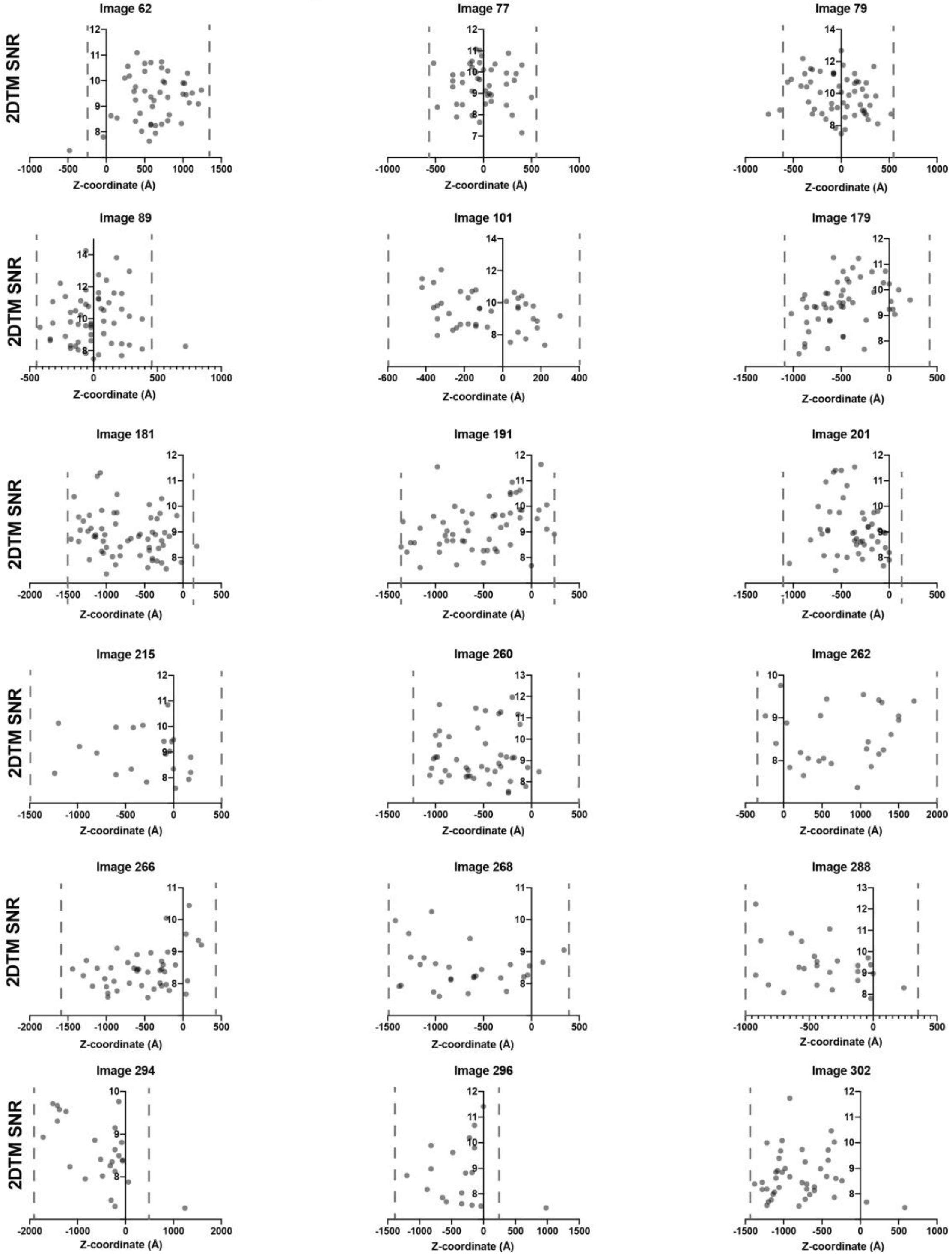

Fig. S5. Scatterplots showing the 2DTM SNRs of LSU-detected targets in images of unmilled *Mycoplasma pneumoniae* cells as a function of z-coordinate relative to the defocus plane at x = 0.

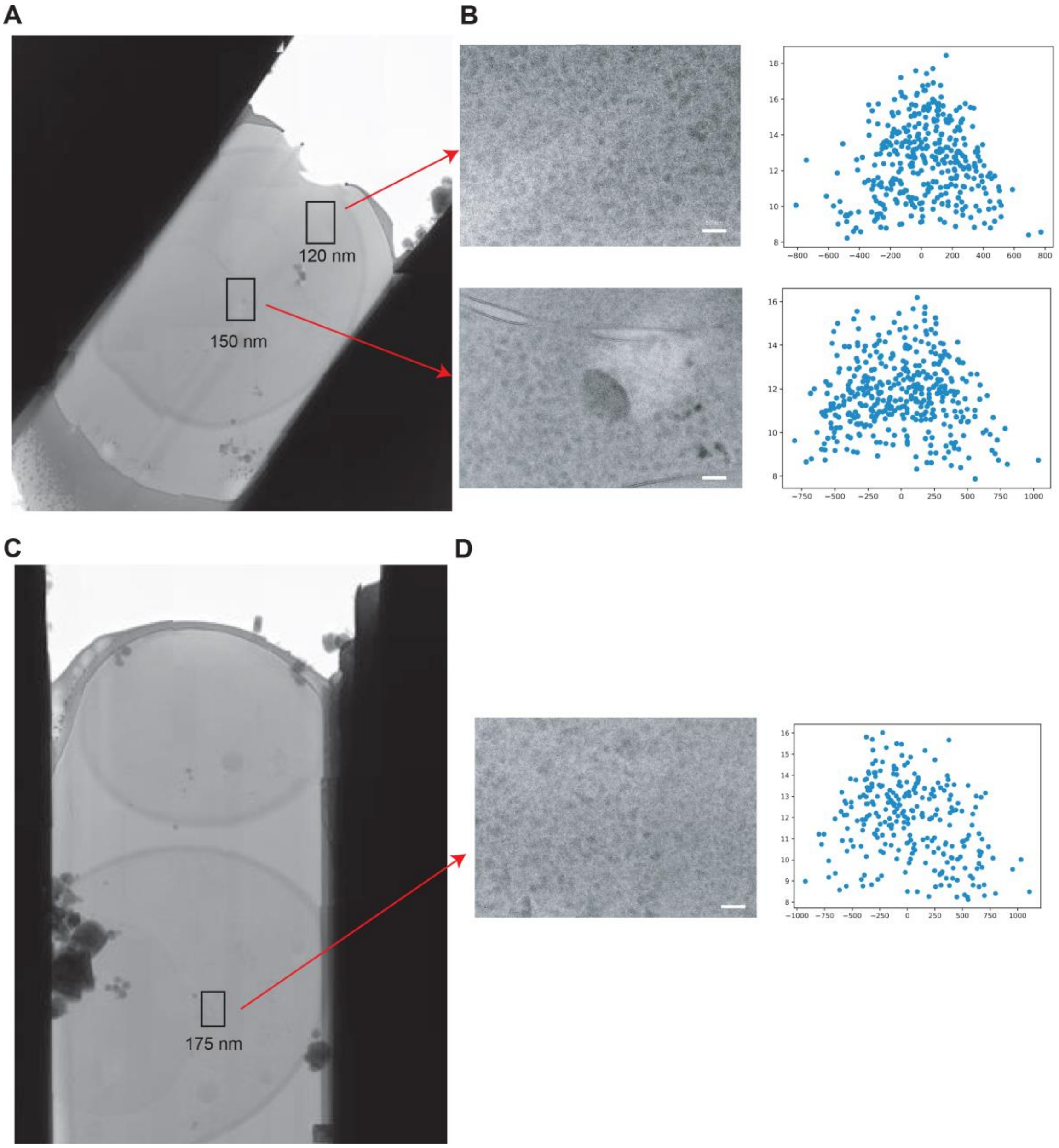

Fig. S6. (A) Image of a lamella. Rectangles designate the regions that the images in (B) were collected from. (B) Left: Images used for 2DTM of the regions of the lamella shown in (A). Scale bars indicate 50 nm. Right: Scatterplots showing the 2DTM SNR of LSUs at the indicated lamella z-coordinates. (C) As in (A), indicating a different lamella. (D) As in (B), showing the image and scatterplot for the region indicated in (C). Scale bars indicate 50 nm.

Table S1. Gaussian fits to z-coordinate bins in Figure 2.

Depth (nm)	Mean	SD	R squared	N		t-test vs 100 nm
**10**	0.8664	0.09367	0.8148		27	P<0.0001
**20**	0.8353	0.09015	0.9243		44	P<0.0001
**30**	0.8733	0.1089	0.9591		161	P<0.0001
**40**	0.9084	0.115	0.9345		245	P<0.0001
**50**	0.9405	0.1167	0.989		383	P<0.0001
**60**	0.9612	0.1193	0.9839		424	P<0.0001
**70**	0.9859	0.1163	0.9644		421	ns
**80**	0.9926	0.1207	0.9703		427	ns
**90**	0.9837	0.1065	0.9676		450	ns
**100**	1.003	0.1079	0.9865		401	_
